# Negative Regulation of RNF90 on RNA Virus-Triggered Antiviral Immune Responses Targeting MAVS

**DOI:** 10.3389/fimmu.2021.730483

**Published:** 2021-08-27

**Authors:** Bo Yang, Ge Zhang, Xiao Qin, Yulu Huang, Xiaowen Ren, Jingliang Sun, Shujun Ma, Yanzi Liu, Di Song, Yue Liu, Yuhan Cui, Hui Wang, Jie Wang

**Affiliations:** ^1^Henan Key Laboratory of Immunology and Targeted Drug, Xinxiang Medical University, Xinxiang, China; ^2^Henan Collaborative Innovation Center of Molecular Diagnosis and Laboratory Medicine, School of Laboratory Medicine, Xinxiang Medical University, Xinxiang, China; ^3^Department of Laboratory Medicine, the Third Affiliated Hospital of Xinxiang Medical University, Xinxiang, China; ^4^Department of Laboratory Medicine, Fuwai Center China Cardiovascular Hospital, Zhengzhou, China

**Keywords:** antiviral innate immune responses, ubiquitination, degradation, signaling pathway, MAVS, RNF90

## Abstract

The antiviral innate immunity is the first line of host defense against viral infection. Mitochondrial antiviral signaling protein (MAVS, also named Cardif/IPS-1/VISA) is a critical protein in RNA virus-induced antiviral signaling pathways. Our previous research suggested that E3 ubiquitin-protein ligases RING-finger protein (RNF90) negatively regulate cellular antiviral responses by targeting STING for degradation, though its role in RNA virus infection remains unknown. This study demonstrated that RNF90 negatively regulated RNA virus-triggered antiviral innate immune responses in RNF90-silenced PMA-THP1 cells, RNF90-deficient cells (including HaCaTs, MEFs, and BMDMs), and RNF90-deficient mice. However, RNF90 regulated RNA virus-triggered antiviral innate immune responses independent of STING. RNF90 promoted K48-linked ubiquitination of MAVS and its proteasome-dependent degradation, leading to the inhibition of innate immune responses. Altogether, our findings suggested a novel function and mechanism of RNF90 in antiviral innate immunity.

## Introduction

RNA viruses, which have RNA as their genetic material, including single-stranded RNA (ssRNA) viruses and double-stranded RNA (dsRNA) viruses, cause many kinds of human infectious diseases (1, 2). A lot of RNA viruses, such as human immunodeficiency virus (HIV), hepatitis C virus (HCV), Ebola virus, Zika virus, respiratory syncytial virus (RSV), influenza viruses, yellow fever virus, dengue virus, severe acute respiratory syndrome (SARS)-associated coronavirus (SARS-CoV), and SARS-CoV-2 are well-known viruses that are infectious and cause serious, even deadly, syndromes in humans (3, 4). The first line to eliminate viral invasions is the host innate immune system (5). The host antiviral immune responses are initiated by the sensing of pathogen-associated molecular patterns (PAMPs) from invading viruses by a series of pattern recognition receptors (PRRs), which results in the production of type I interferons (IFNs) and other cytokines or chemokines essential to host antiviral responses (6).

For RNA viruses, the viral nucleic acids are critical PAMPs, and the viral RNAs are recognized by TLR3 or RIG-I-like receptors (RLRs) (7). In most cell types, two RLRs, retinoic-acid-inducible protein I (RIG-I) and Melanoma differentiation-associated gene 5 (Mda5), serve as cytosolic RNA receptors (8). Generally speaking, RIG-I is responsible for the production of type I IFNs and proinflammatory cytokines upon the infection by Newcastle disease virus (NDV), vesicular stomatitis virus (VSV), influenza virus, and Sendai virus (SeV), and Japanese encephalitis virus (JEV), whereas Mda5 was required for the responses against encephalomyocarditis virus (EMCV) and *in vitro* transcribed poly I:C (9).

Activated RIG-I and MDA5 by dsRNA engagement interact with the adaptor protein mitochondrial antiviral-signaling protein (MAVS), which is also known as caspase-activation recruitment-domain adaptor inducing I FN-β (Cardif), IFN-β promoter stimulator (IPS-1), or virus-induced signaling adaptor (VISA), *via* a caspase recruitment domain (CARD)-CARD interaction, and triggers MAVS mediating signaling pathways, including the recruitment of downstream tumor necrosis factor (TNF) receptor-associated factor (TRAF) family members, the TRAF family member-associated NF-κB activator (TANK)-binding kinase 1 (TBK1) and inducible IκB kinase (IKK-i or IKK-ϵ), leading to the activation of the transcription factor NF-κB and IRF3 (10–14). Thus, MAVS is critical to innate immunity and serves as the center of the antiviral innate immune responses against RNA viruses.

The host immune signaling pathways must be tightly controlled because its excessive activation may cause tissue damage and contribute to the pathogenesis of many human diseases (15–17). Accumulating evidence suggests that ubiquitination is part of this exquisite regulatory system and adjust the strength and duration of the immune responses in a timely and efficient way (18). Ubiquitin has seven Lys residues (K6, K11, K27, K29, K33, K48, and K63) and can be linked to its target proteins to regulate their functions by various mechanisms (19). K48-linked ubiquitination is mainly associated with proteasome-mediated degradation of proteins, whereas K63-linked modification usually regulates signaling pathways in a non-proteolytic manner (20). The process of ubiquitination was catalyzed sequentially by three different types of enzymes, E1 ubiquitin-activating enzyme, E2 ubiquitin-conjugating enzymes, and E3 ubiquitin-protein ligases (21). Human genome contains more than 600 E3 ubiquitin-protein ligases, responsible for the high selection of target proteins, including HECT-, RING- and RBP-type E3 ubiquitin-protein ligases (22).

As the center adaptor protein in the RLR signaling pathways, MAVS has been reported to be targeted by several E3 ubiquitin-protein ligases for the regulation of antiviral immune responses (23). For example, TRIM25 catalyzed K48-linked ubiquitination of MAVS and marked it for proteasomal degradation (24), whereas TRIM31 catalyzes the K63-linked polyubiquitination of MAVS and promotes its activation and subsequent induction of type I IFNs (25). In addition, our previous research suggested that TRIM44 stabilized MAVS by inhibiting the K48-linked ubiquitination and degradation of MAVS (26).

RNF90 (also called TRIM7/GNIP), was identified as a RING-type E3 ubiquitin-protein ligase in various cancer pathological conditions (27–30). In innate immunity, RNF90 positively regulates the TLR4-mediated innate response *via* its E3 ligase domain in macrophages (31). RNF90 has been reported to regulate norovirus replication (32). A recent study demonstrated that RNF90 inhibited enterovirus replication by targeting viral 2BC protein for ubiquitination and degradation (33). Our previous study indicated RNF90 deficiency protected mice from DNA virus infection and RNF90 negatively modulated DNA virus- or cytosolic DNA-triggered signaling pathway *via* the enhancement of K48-linked ubiquitination of STING and its proteasome-dependent degradation (34). A recent study showed that RNF90 promoted the Lys63 (K63)-linked polyubiquitination of the envelope protein of Zika virus, which enhances virus entry in cells and brain tissue *in vivo* (35). In this research, we investigated the role of RNF90 in RNA virus-triggered signaling pathways, and our findings suggested RNF90 as a negative regulator of the RIG-I mediated signaling pathway. RNF90 was induced during RNA virus infection, interacted with MAVS, and promoted the K48-linked ubiquitination and subsequent proteasome-dependent degradation of MAVS. RNF90 deficiency enhanced RNA virus-triggered innate immune responses and protected mice from RNA virus infection. Thus, our study demonstrated that RNF90 promoted MAVS degradation and suggest a novel mechanism that regulates antiviral immune responses of host cells against RNA virus invasion.

## Materials and Methods

### Mice

We generated RNF90-deficient mice as described previously (34). The sequences of sgRNA were: K1 (GGCGGAGTTCCAAGCGCTGCGGG), K2 (GGGTCGGCTTCTAAGCCGACTGG) and K3 (CTGGATCTGGCCGCTGAGTTTGG). No RNF90 mRNA or truncated proteins were detected in the RNF90-deficient mice. Mice were housed in a facility with access to food and water and were maintained under a 12-h light/12-h dark cycle. All animal procedures were performed according to guidelines approved by the committee on animal care at Xinxiang Medical University, China. The age- and sex-matched wildtype (WT) and RNF90-deficient mice were used in the experiments.

### Plasmids

Plasmids encoding human RNF90 or its deletion mutants were constructed as described previously (34). HA-Ubi, HA-K48-Ubi, HA-K63-Ubi, HA-K48R-Ubi, HA-K63R-Ubi, pIFN-β-Luc, HA/Flag-MAVS were obtained as described previously (26). HA-K6-Ubi (22900), HA-K11-Ubi (22901), HA-K27-Ubi (22902), HA-K29-Ubi (22903), HA-K33-Ubi (17607) were purchased from Addgene.

### Reagents

The antibodies for immunoblot analysis or immunoprecipitation were listed as follows: anti-Flag (F3165, Sigma-Aldrich), anti-HA (901515, Biolend), anti-Myc (66004-1-Ig, Proteintech), anti-RNF90 (sc-109107, Santa Cruz; ab170538, Abcam), anti-MAVS (14341-1-AP, Proteintech), anti-p-TBK1 (5483T, Cell Signaling Technology), anti-TBK1 (CSB-PA024154LA01HU, Flarbio), anti-p-IRF3 (4947, Cell Signaling Technology), anti-IRF3 (sc-9082, Santa Cruz), anti-p-p65 (3033, Cell Signaling Technology), anti-p65 (10745-1-AP, Proteintech), anti-STING (19851-1-AP, Proteintech), anti-Ubi (sc-8017, Santa Cruz), anti-Ubi-K48 (05-1307, Millipore), anti-Ubi-K63 (05-1313, Millipore), anti-VSVg (YM3006, Immunoway), anti-H3 (CSB-PA010109LA01HU, Flarbio), anti-β-tubulin(10068-1-AP, Proteintech), and anti-β-actin (60008-1, Proteintech). The poly(I:C) (tlrl-picw), HSV60 (tlrl-hsv60n) and cGAMP (tlrl-nacga23) were obtained from InvivoGen. The PMA (S1819) was obtained from Beyotime Biotechnology. MG132 (474790) was purchased from Millipore. 3-MA (M9281) and NH4Cl (A9434) were purchased from Sigma-Aldrich.

### Cell Culture

HaCaT keratinocytes were obtained from Procell Life Science & Technology Co., Ltd., (Wuhan, China). HEK293T and THP1 cells were purchased from Stem Cell Bank, Chinese Academy of Sciences. The procedures for the generation of BMDMs and MEFs have been described previously (36, 37). THP1 STING-KO cells (thpd-kostg) were purchased from InvivoGen. RNF90-deficient HaCaT cells were generated by the Laboratory of Genetic Regulators in the Immune System in Xinxiang Medical University through CRISPR/Cas9-mediated gene editing. THP1 cells were cultured in RPMI 1640, whereas HaCaT and HEK293T cells were grown in Dulbecco’s modified Eagle's medium (DMEM). Phorbol-12-myristate-13-acetate (PMA)-differentiated THP1 (PMA-THP1, a human macrophage-like cell line) cells referred to THP1 cells that were pretreated with 100ng/ml PMA for 24 h. All cells were supplemented with 10% FBS (Gibco), 4 mM L-glutamine, 100μg/ml penicillin, and 100U/ml streptomycin under humidified conditions with 5% CO_2_ at 37°C. Transfection of HaCaT, HEK293T, THP1, MEFs, and BMDMs was performed with Lipofectamine 2000 (Invitrogen) according to the manufacturer’s instructions.

### Immunoprecipitation and Immunoblot Analysis

Immunoprecipitation and immunoblot analysis were performed as described previously (38). Briefly, cells were lysed in lysis buffer (1.0% Nonidet P-40, 20 mM Tris-HCl, 10% glycerol, 150 mM NaCl, 0.2 mM Na_3_VO_4_, 1mM NaF, 0.1 mM sodium pyrophosphate with a protease inhibitor ‘cocktail’ (Roche), pH 8.0). After centrifugation for 20 min at 14,000g, supernatants were collected and incubated with the indicated antibodies together with protein A/G Plus-agarose immunoprecipitation reagent (sc-2003, Santa Cruz) at 4°C for 3 h or overnight. After three washes, the immunoprecipitates were boiled in an SDS sample buffer for 10 min and analyzed by immunoblot.

### Nuclear Extracts

The nuclear extracts were prepared as described previously (39). In short, cells were lysed with fresh buffer A (10 mM HEPES, 1.5 mM MgCl2 · 6 H2O, 10 mM KCl, 0.5 mM DTT, 0.1% Nonidet P-40, with a protease inhibitor ‘cocktail’ (Roche), pH 7.9). The lysate was placed on ice for 10 min and centrifuged at 10,000 rpm for 5 min at 4°C to remove cytoplasmic proteins. Nuclear proteins were extracted from the pellet in ice-cold fresh buffer C (20 mM HEPES, 1.5 mM MgCl2 · 6 H2O, 0.42 M NaCl, 0.2 mM EDTA, 25% glycerol, 0.5 mM DTT, with a protease inhibitor ‘cocktail’ (Roche), pH 7.9). Insoluble material was removed by centrifugation at 10,000 rpm for 5 min at 4°C. Protein concentration was measured by BCA protein assay reagent kit.

### Real-Time PCR

Total RNA was extracted from the cultured cells with TRIzol reagent (Invitrogen). All gene transcripts were quantified by real-time PCR with SYBR Green qPCR Master Mix using a 7500 Fast real-time PCR system (Applied Biosystems). The relative fold induction was calculated using the 2^-△△Ct^ method. The primers used for real-time PCR were as follows:

Human IFN-β,
Forward, 5’- CACGACAGCTCTTTCCATGA -3’;
Reverse, 5’- AGCCAGTGCTCGATGAATCT -3’
Human CXCL10,
Forward, 5’- GGTGAGAAGAGATGTCTGAATCC -3’;
Reverse, 5’- GTCCATCCTTGGAAGCACTGCA -3’
Human TNF-α,
Forward, 5’- GGCGTGGAGCTGAGAGATAAC -3’;
Reverse, 5’- GGTGTGGGTGAGGAGCACAT -3’
Human RANTES,
Forward, 5’- TACACCAGTGGCAAGTGCTC -3’;
Reverse, 5’- ACACACTTGGCGGTTCTTTC -3’
Human ISG56,
Forward, 5’- GCCATTTTCTTTGCTTCCCCTA -3’;
Reverse, 5’- TGCCCTTTTGTAGCCTCCTTG -3’
Human GAPDH,
Forward, 5’-TCAACGACCACTTTGTCAAGCTCA-3’;
Reverse, 5’-GCTGGTGGTCCAGGTCTTACT-3’
Mouse IFN-β,
Forward, 5’- TCCTGCTGTGCTTCTCCACCACA -3’;
Reverse, 5’- AAGTCCGCCCTGTAGGTGAGGTT -3’
Mouse CXCL10,
Forward, 5’- ATCATCCCTGCGAGCCTATCCT -3’;
Reverse, 5’- GACCTTTTTTGGCTAAACGCTTTC -3’
Mouse TNF-α,
Forward, 5’- CGTAGGCGATTACAGTCACGG -3’;
Reverse, 5’- GACCAGGCTGTCGCTACATCA -3’
Mouse ISG56,
Forward, 5’-ACAGCAACCATGGGAGAGAATGCTG-3’;
Forward, 5’-ACGTAGGCCAGGAGGTTGTGCAT-3’
Mouse GAPDH,
Forward, 5’- ACGGCCGCATCTTCTTGTGCA-3’;
Reverse, 5’- ACGGCCAAATCCGTTCACACC-3’.

### ELISA

The culture media of BMDMs and MEFs or the serum of mice were collected for measurement of IFN-β (PBL) and TNF-α (Thermo Fisher Scientific) by ELISA according to the manufacturer’s instructions.

### RNA Interference

RNF90 Stealth-RNAi siRNA was designed by the Invitrogen BLOCKiT RNAi Designer. The small interfering RNA (siRNA) sequences used were as follows:

R2,
Forward, 5’-GAGGACUGUGAGGUGUUCCGGUCCA-3’;
Reverse, 5’-UGGACCGGAACACCUCACAGUCCUC -3’
R3,
Forward, 5’-CAGUCUCUUCUGAGAUGAAGAAUAA-3’;
Reverse, 5’-UUAUUCUUCAUCUCAGAAGAGACUG -3’

The Silencer Select negative control siRNA was obtained from Invitrogen (Catalog no.4390843). Lipofectamine 2000 was used for the transfection of PMA-THP1 or HaCaT cells with siRNA.

### Viruses and Infection

Cells were infected with VSV (MOI=1) for 1.5 h. Then the cells were washed with PBS and cultured in fresh media. For the *in vivo* study, age- and sex-matched mice were intravenously or intraperitoneally infected with VSV. VSV viral titer was determined by the plaque-forming assay on Vero cells.

### *In Vitro* Ubiquitination Assay

MAVS, RNF90, and RNF90 mutants were expressed with a TNT Quick-coupled Transcription/Translation Systems kit (L1171, Promega). *In vitro* ubiquitination assay was performed with a ubiquitination kit (BML-UW9920, Enzo Life Science) following the manufacturer’s instructions.

### Luciferase Reporter Gene Assay

Luciferase reporter gene assays were performed as described previously (38). In short, HEK293T cells were transfected with indicated plasmids. 24 h after transfection, cells were lysed, and reporter activity was analyzed with the Dual-Luciferase Reporter Assay System (Promega).

### Confocal Microscopy

After treatment, HEK293T cells were fixed with 4% PFA in PBS, permeabilized with Triton X-100, and then blocked with 1% BSA in PBS. Nuclei were stained with 4, 6-diamidino-2-phenylindole (DAPI).

### Statistics

The data are presented as the means ± SD from at least three independent experiments. The statistical comparisons between the different treatments were performed using the unpaired Student *t*-test, and *P* < 0.05 was considered statistically significant.

## Results

### RNF90 Negatively Regulates RNA Virus-Induced Innate Immune Responses

**Figure 1:**
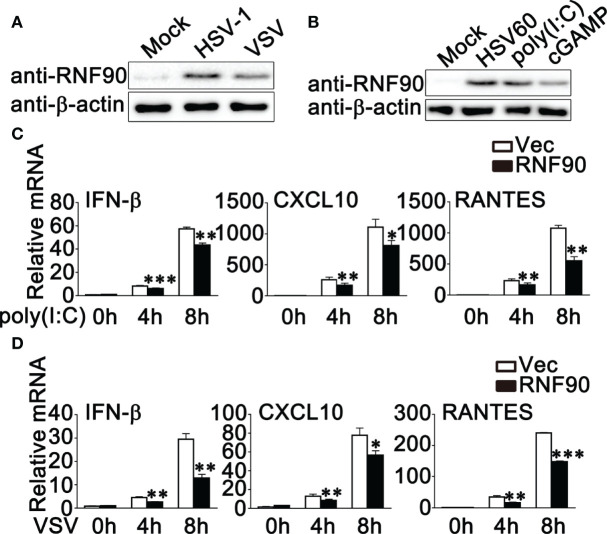
RNF90 overexpression inhibits RNA virus-triggered innate immune responses. **(A)** PMA-THP1 cells were stimulated with HSV-1 (MOI = 1) or VSV (MOI = 1) for 8 h. Afterwards, the cells were lysed for immunoblot assays. **(B)** PMA-THP1 cells were transfected with 1 μg/ml HSV60, 2.5 μg/ml poly(I:C), or 1 μg/ml cGAMP **(B)** for 8 h. Afterwards, the cells were lysed for immunoblot assays. **(C, D)** HEK293T cells were transfected with an empty vector (Vec) or RNF90 plasmid. At 24 h after transfection, HEK293T cells were treated with poly(I:C) (2.5 μg/ml) **(C)** or VSV (MOI = 1) **(D)** for indicated time periods. Then the cells were lysed for real-time PCR analysis. β-actin was used as a loading control in all the immunoblot assays. The data are representative of three independent experiments and are presented as mean ± SEM. *P < 0.05, **P < 0.01, ***P < 0.001.

**Figure 2:**
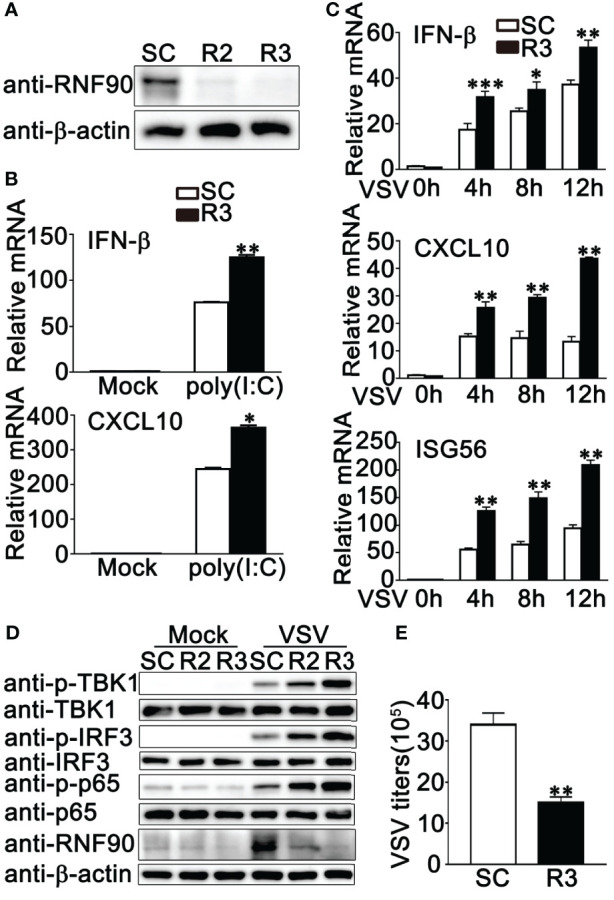
RNF90 knockdown promotes RNA virus-triggered innate immune responses. **(A)** PMA-THP1 cells were transfected with control siRNA (SC) or RNF90-specific siRNA (R2 and R3). At 24 h after transfection, the cells were infected with VSV (MOI = 1) for 8 h and then lysed for immunoblot assay. **(B, C)** PMA-THP1 cells were transfected with control siRNA (SC) or RNF90-specific siRNA (R3). 24 h later, PMA-THP1 cells were treated with poly(I:C) (2.5 μg/ml) for 8 h **(B)** or VSV (MOI = 1) **(C)** for indicated time periods. Then the cells were lysed for real-time PCR analysis. **(D)** PMA-THP1 cells were transfected with control siRNA (SC) or RNF90-specific siRNA (R2, R3). At 24 h after transfection, the cells were infected with VSV (MOI = 1) for 8 h. Then, the cells were lysed for immunoblot analysis. **(E)** PMA-THP1 cells were transfected with control siRNA (SC) or RNF90-specific siRNA (R3). At 24 h after transfection, the cells were infected with VSV (MOI = 1) for 24 h. The titers of VSV were determined by standard plaque assay. β-actin was used as a loading control in all the immunoblot assays. The data are representative of three independent experiments and are presented as mean ± SEM. *P < 0.05, **P < 0.01, ***P < 0.001.

Our previous work indicated that RNF90 negatively regulated DNA virus- or cytosolic DNA-triggered antiviral innate immune responses (34). We wondered whether RNF90 has a role in RNA virus-triggered signaling pathways. To address this issue, firstly, we examined the expression pattern of RNF90 upon poly (I:C) transfection or RNA virus infection. As shown in **Figures 1A, B**, immunoblot results indicated that RNF90 expression was induced in PMA-THP1 cells upon VSV infection (**Figure 1A**) or poly (I:C) transfection (**Figure 1B**). Then, we evaluated the effect of RNF90 on RNA virus-induced immune responses. Induction of IFN-β, CXCL10, and RANTES upon poly (I:C) transfection or VSV infection was inhibited by RNF90 overexpression in mRNA levels (**Figures 1C, D**). Next, we used the knockdown approach to further address endogenous RNF90 in RNA virus-triggered innate immune responses. PMA-THP1 cells were transfected with control siRNA (SC) or two pairs of siRNA oligonucleotides specific for RNF90 RNA (R2, R3). As shown in **Figure 2A**, both R2 and R3 inhibited endogenous RNF90 expression. In PMA-THP1 cells, real-time PCR results indicated the production of IFN-β and CXCL10 was increased in RNF90-silenced PMA-THP1 cells compared to SC-transfected cells upon poly (I:C) transfection (**Figure 2B**). Consistently, RNF90 knockdown promoted VSV-induced antiviral immune responses, including the production of IFN-β, CXCL10, and RANTES (**Figure 2C**) and the phosphorylation of TBK1, IRF3, and p65 (**Figure 2D**). In addition, standard plaque assay was performed to analyze virus titers in the cell supernatants. The results indicated RNF90 knockdown decreased VSV titers (**Figure 2E**), suggesting the VSV-triggered antiviral immune responses was inhibited by RNF90. Altogether, our results suggested a negative regulatory role of RNF90 in RNA virus-induced innate immune responses.

### RNF90 Deficiency Promotes RNA Virus-Triggered Innate Immune Responses in HaCaT Cells

**Figure 3:**
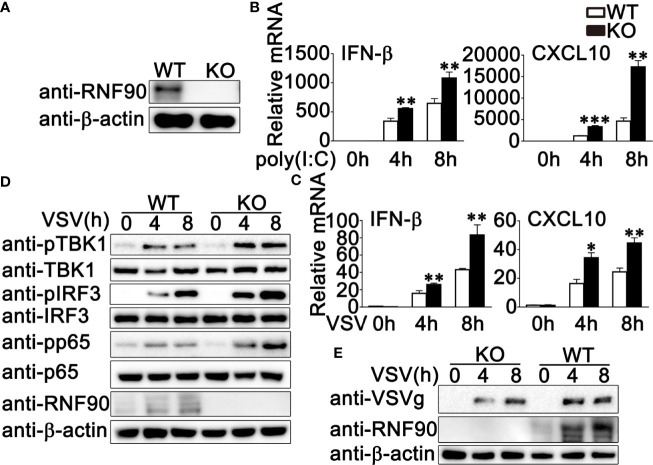
RNF90 deficiency promotes RNA virus-triggered innate immune responses in HaCaT cells. **(A)** WT and RNF90-deficient (KO) HaCaT cells were lysed for immunoblot analysis. **(B, C)** WT and RNF90-deficient (KO) HaCaT cells were treated with poly (I:C) (2.5 μg/ml) **(B)** or VSV (MOI = 1) **(C)** for indicated time points and then lysed for real-time PCR analysis. **(D)** WT and RNF90-deficient (KO) HaCaT cells were infected with VSV (MOI = 1) for indicated time points and then lysed for immunoblot analysis. **(E)** WT and RNF90-deficient (KO) HaCaT were infected with VSV (MOI = 1) for indicated time points and then lysed for immunoblot analysis. β-actin served as a loading control in all the immunoblot assays. The data are representative of three independent experiments. *P < 0.05, **P < 0.01, ***P < 0.001.

To further investigate the role of RNF90 in RNA virus infection, we generated the RNF90-deficient HaCaT cell line by CRISPR/Cas9 strategy. As shown in **Figure 3A**, the expression of RNF90 could not be detected in RNF90-deficient HaCaT cells by immunoblot assays. Then we stimulated the RNF90-deficient HaCaT cells and WT HaCaT cells with poly (I:C) transfection or VSV infection and evaluated the effects of RNF90 on RNA virus-triggered immune responses. Real-time PCR assays indicated that, compared to WT HaCaT cells, RNF90-deficient HaCaT cells exhibited higher IFN-β and CXCL10 production upon poly (I:C) transfection or VSV infection (**Figures 3B, C**). In addition, in RNF90-deficient HaCaT cells, the phosphorylation of TBK1, IRF3, and p65 was higher than that in WT HaCaT cells (**Figure 3D**). Finally, immunoblot results demonstrated decreased virus protein VSVg in RNF90-deficient HaCaT cells (**Figure 3E**). Altogether, our findings suggested RNF90 deficiency in HaCaT cells enhanced RNA virus-triggered innate immune responses, suggesting the inhibitory role of RNF90 in RNA virus-induced signaling pathways.

### RNF90 Deficiency Promotes RNA Virus-Triggered Innate Immune Responses in Primary MEFs and BMDMs

**Figure 4:**
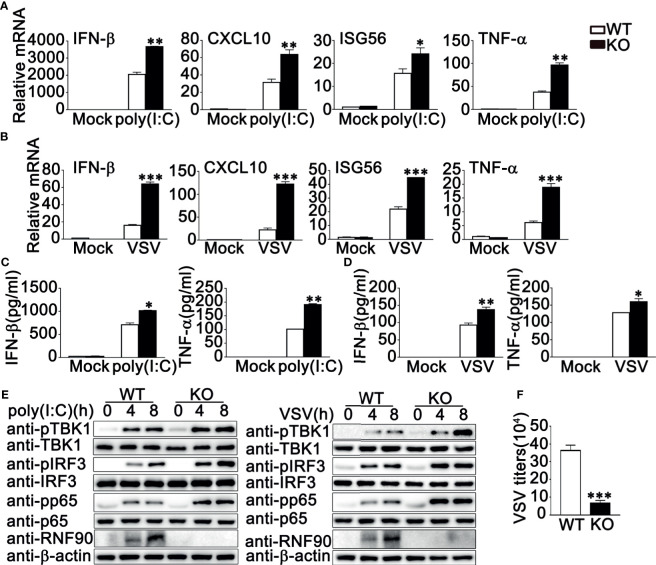
RNF90 deficiency promotes RNA virus-triggered innate immune responses in MEFs. **(A, B)** WT and RNF90-deficient (KO) MEFs were treated with poly(I:C) (2.5 μg/ml) **(A)** or VSV (MOI = 1) **(B)** for 8 h. The cells were lysed for real-time PCR analysis. **(C, D)** WT and RNF90-deficient (KO) MEFs were stimulated with poly(I:C) (2.5 μg/ml) **(C)** or VSV (MOI = 1) **(D)** for 24 h. The supernatants were collected and subjected to ELISA analysis. **(E)** WT and RNF90-deficient (KO) MEFs were transfected with poly (I:C) (2.5 μg/ml) or VSV (MOI = 1) for indicated time points. Then the cells were lysed for immunoblot analysis. **(F)** WT and RNF90-deficient (KO) MEFs were infected with VSV for 24 h. The titers of VSV were determined by standard plaque assays. β-actin served as a loading control in all the immunoblot assays. The data are representative of three independent experiments and are presented as mean ± SEM. *P < 0.05, **P < 0.01, ***P < 0.001.

**Figure 5:**
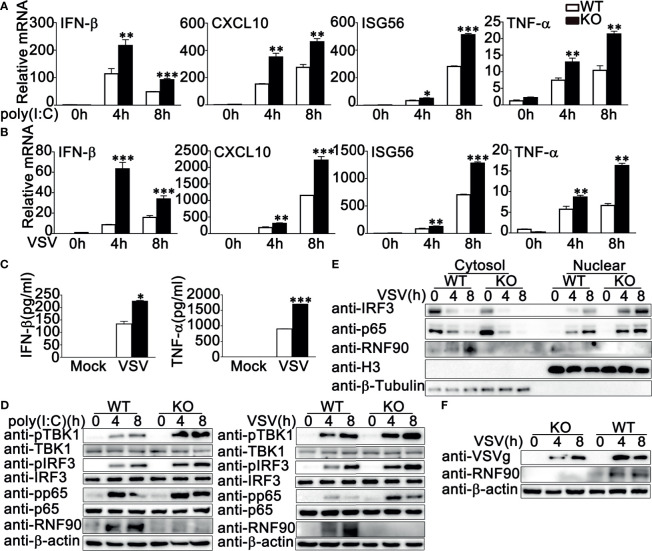
RNF90 deficiency promotes RNA virus-triggered innate immune responses in BMDMs. **(A, B)** WT and RNF90-deficient (KO) BMDMs were treated with poly(I:C) (2.5 μg/ml) **(A)** or VSV (MOI = 1) **(B)** for indicated time periods. Then the cells were lysed for real-time PCR analysis. **(C)** WT and RNF90-deficient (KO) BMDMs were infected with VSV (MOI = 1) for 24 h. The supernatants were collected and subjected to ELISA analysis. **(D)** WT and RNF90-deficient (KO) BMDMs were treated with poly(I:C) (2.5 μg/ml) or VSV (MOI = 1) for indicated time periods. Then the cells were lysed for immunoblot analysis. **(E)** WT and RNF90-deficient (KO) BMDMs were infected with VSV (MOI = 1) for indicated time points and then fractionated into cytosolic and nuclear subfractions. The immunoblot assay was performed as indicated. **(F)** WT and RNF90-deficient (KO) BMDMs were infected with VSV (MOI = 1) for indicated time points. Then the cells were lysed for immunoblot analysis. β-actin served as a loading control in all the immunoblot assays. The data are representative of three independent experiments. *P < 0.05, **P < 0.01, ***P < 0.001.

Next, we isolated and cultured the primary MEFs and BMDMs from RNF90-deficient mice and examined the effects of RNF90 deficiency on RNA virus-triggered antiviral immune responses in primary non-immune and immune cells. As shown in **Figures 4A, B**, RNF90 deficiency enhanced the production of IFN-β, CXCL10, ISG56, and TNF-α in mRNA levels in MEFs upon the treatment of poly (I:C) transfection or VSV infection. Similar results were obtained from BMDMs isolated from RNF90-deficient mice (**Figures 5A, B**). The increase of IFN-β and ISG56 in RNF90-deficient MEFs upon VSV infection could be blocked by RNF90 transfection (**Figure S1**). Additionally, ELISA assays confirmed the increase of IFN-β, and TNF-α production in protein levels in RNF90-deficient MEFs, compared to WT MEFs, upon the treatment of poly (I:C) transfection (**Figure 4C**). Consistently, compared to the cells isolated from WT mice, RNF90-deficient MEFs and BMDMs exhibited a higher IFN-β and TNF-α production after VSV infection (**Figures 4D**, **5C**). Furthermore, in MEFs and BMDMs isolated from WT mice, the impairment of RNF90 resulted in enhanced phosphorylation of TBK1, IRF3, and p65 upon poly (I:C) transfection or VSV infection (**Figures 4E**, **5D**). VSV triggered the nuclear accumulation of IRF3, and p65 was observed in BMDMs (**Figure 5E**). In BMDMs isolated from RNF-deficient mice, the VSV-triggered nuclear accumulation of IRF3 and p65 was enhanced (**Figure 5E**). VSV infection was also suppressed by RNF90 deficiency as suggested by VSV titers using plaque assays in MEFs and VSV protein VSVg expression using immunoblot assays in BMDMs (**Figures 4F**, **5F**). Altogether, our findings in RNF90-deficient primary non-immune and immune cells confirmed that RNF90 inhibited RNA virus-triggered innate immune responses.

### RNF90 Deficiency Protects Mice From RNA Virus Infection

**Figure 6:**
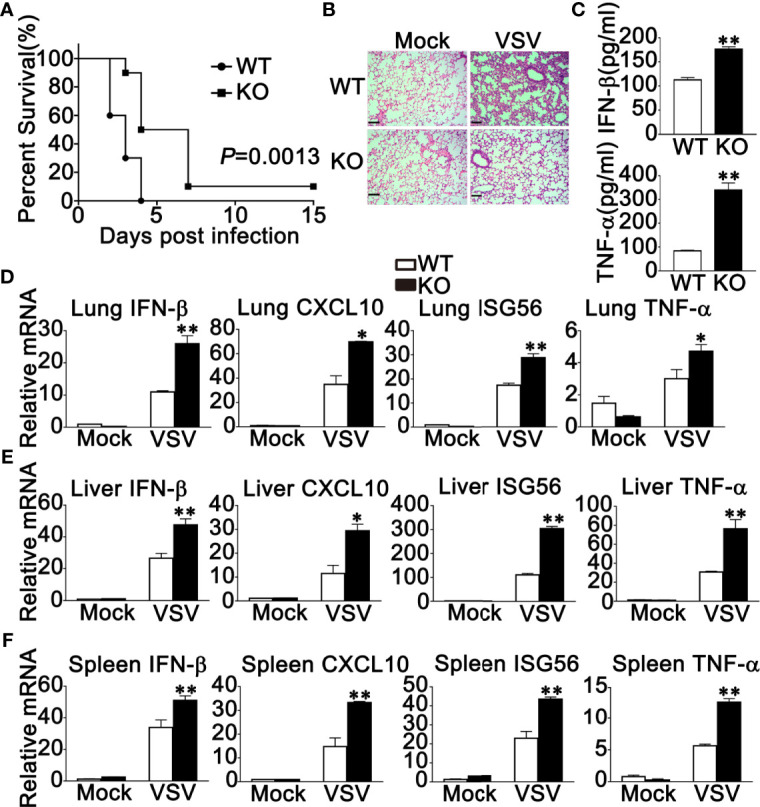
RNF90 deficiency protects mice from RNA virus infection. **(A)** Sex and age-matched WT and RNF90-deficient (KO) mice (n = 5) were intraperitoneally (i.p.) infected with VSV [5×10^8^ plaque-forming units (PFU)]. Mouse survival rates were observed and recorded for 15 days. The data were analyzed by Log-rank (Mantel-Cox) test. **(B)** Sex and age-matched WT and RNF90-deficient (KO) mice were intraperitoneally (i.p.) infected with VSV (5×10^7^ PFU) for 24 h, and lung sections were analyzed by H&E staining. Scale bars, 200 μm. **(C)** ELISA of IFN-β and TNF-α in serum of WT and RNF90-deficient (KO) mice 6 h after intraperitoneal (i.p.) infection with VSV (5×10^7^ PFU). **(D-F)** WT and RNF90-deficient (KO) mice were intraperitoneally infected with VSV (5×10^7^ PFU) for 24 h, and then the lungs **(D)**, livers **(E)**, and spleens **(F)** of the mice were subjected to real-time PCR analysis. The data are representative of three independent experiments. *P < 0.05, **P < 0.01.

Next, WT and RNF90-deficient mice were used to investigate the role of RNF90 in antiviral immune responses against VSV infection *in vivo*. RNF90-deficient mice exhibited prolonged survival after VSV infection, suggesting the protective role of RNF90 impairment during VSV infection in mice (**Figure 6A**). Additionally, less lung destruction was observed in the lungs from RNF90-deficient mice than that from WT mice after VSV infection (**Figure 6B**). Compared to WT mice, higher IFN-β and TNF-α expression levels were observed in the serum of RNF90-deficient mice upon VSV infection (**Figure 6C**). Next, we evaluated the antiviral immune responses in different organs of mice. As shown in **Figures 6D–F**, in lung, liver, and spleen, RNF90 impairment significantly promoted VSV-triggered antiviral immune responses, as suggested by the increased production of IFN-β, CXCL10, ISG56, and TNF-α. Altogether, our findings suggested that RNF90 deficiency protected mice from RNA virus infection.

### The Effect of RNF90 on RNA Virus Infection Is Independent of STING

**Figure 7:**
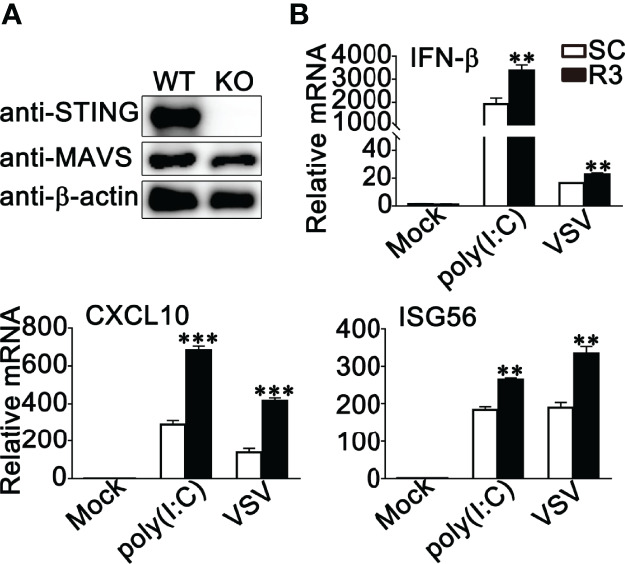
RNF90 inhibits RNA virus-triggered innate immune responses independent of STING Signaling. **(A)** WT and STING-deficient (KO) PMA-THP1 cells were lysed for immunoblot analysis. β-actin served as a loading control. **(B)** STING-deficient (KO) PMA-THP1 cells were transfected with control siRNA (SC) or RNF90-specific siRNA (R3) for 24 h and then transfected with poly (I:C) or infected with VSV for 8 h. Then, the cells were lysed for real-time PCR assays. The data are representative of three independent experiments and are presented as mean ± SEM. **P < 0.01, ***P < 0.001.

STING has been reported to play a very important role in both RNA viruses- and DNA viruses-induced antiviral immune responses (40–43). Our previous study clarified that RNF90 negatively regulated DNA virus- or cytosolic DNA-triggered antiviral immune responses by targeting STING for degradation (34). Therefore, we wondered whether the effect of RNF90 on RNA virus-induced signaling pathways is independent of STING. To address this question, we constructed STING-deficient THP1 cells and examined the role of RNF90 in RNA virus-triggered immune responses in the absence of STING. As is shown in **Figures 7A**, **S2**, STING-deficient THP1 cells showed undetectable STING expression and impaired IFN-β production upon poly(dA:dT) or HSV60 transfection. Then, control siRNA (SC) or RNF90-specific siRNA (R3) were transfected into STING-deficient PMA-THP1 cells (**Figure 7B**). Real-time PCR assays indicated RNF90 knockdown promoted the production of IFN-β, CXCL10, and ISG56 upon poly (I:C) transfection or VSV infection in the absence of STING, suggesting RNF90 negatively regulated RNA virus-triggered immune responses targeting other proteins involved in the RLR signaling pathway. Altogether, our findings suggested that the effect of RNF90 on RNA virus-triggered antiviral immune response is independent of STING.

### RNF90 Interacts With MAVS

**Figure 8:**
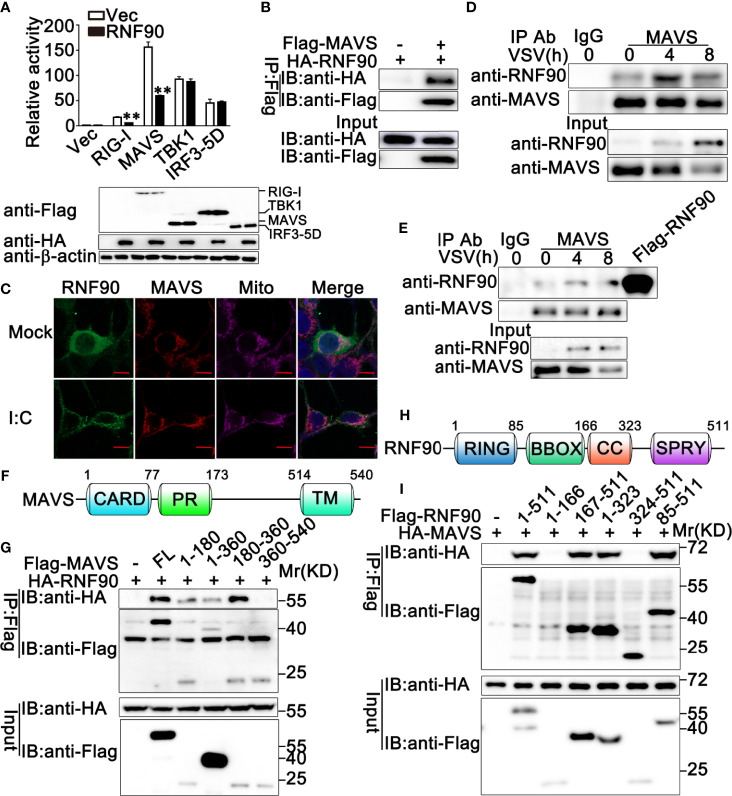
RNF90 interacts with MAVS. **(A)** HEK293T cells were transfected with an IFN-β luciferase reporter, together with RIG-I, MAVS, TBK1, or IRF3-5D and the empty vector (Vec) or the RNF90 plasmid. At 24h after transfection, the cells were lysed for luciferase assay and immunoblot analysis. β-actin served as a loading control. **P < 0.01. **(B)** HEK293T cells were transfected with HA-RNF90, together with the empty vector (-) or Flag-MAVS plasmid. At 24 h after transfection, the cells were lysed and subjected to immunoprecipitation (IP) and immunoblot (IB) analysis. **(C)** HEK293T cells were transfected with Flag-RNF90, at 24 h after transfection, HEK293T cells were stimulated with poly(I:C) (2.5 μg/ml) or left untreated for another 8 h. Immunofluorescence was performed using anti-Flag (green) and anti-MAVS (red). Mitochondria and Nuclei were stained with Mito Tracker or DAPI, respectively. Scale bars, 10 μm. **(D, E)** PMA-THP1 cells **(D)** or HaCaT cells **(E)** were infected with VSV (MOI = 1) for indicated time points, and then the cell lysates were subjected to immunoprecipitation (IP) and immunoblot (IB) analysis as indicated. **(F)** A schematic presentation of full-length MAVS and its mutants. **(G)** HEK293T cells were transfected with plasmids as indicated. At 24 h after transfection, the cell lysates were subjected to immunoprecipitation (IP) and immunoblot (IB) analysis as indicated. **(H)** A schematic presentation of full-length RNF90 and its mutants. **(I)** HEK293T cells were transfected with plasmids as indicated. At 24 h after transfection, the cell lysates were subjected to immunoprecipitation (IP) and immunoblot (IB) analysis as indicated. The data are representative of three independent experiments.

To explore the molecular mechanisms underlying the negative regulatory role of RNF90 in RNA virus-triggered anti-viral immune responses, we first identified the target protein of RNF90 by luciferase assay. RIG-I, MAVS, TBK1, and IRF3-5D (the constitutively active mutant of IRF3) are essential to the VSV-induced antiviral signaling pathways. Thus, we examined the effects of RNF90 overexpression on IFN-β reporter activation induced by these molecules. As shown in **Figure 8A**, RNF90 overexpression inhibited IFN-β reporter activation induced by RIG-I, and MAVS, but not by TBK1 and IRF3-5D. Thus, we hypothesized that RNF90 might target MAVS to inhibit RIG-I signaling. To address this hypothesis, we investigated whether RNF90 interacted with MAVS. HA-RNF90 and Flag-MAVS were co-transfected into HEK293T cells, and co-immunoprecipitation assays revealed that RNF90 interacted with MAVS (**Figure 8B**). Additionally, confocal microscopy indicated that Flag-RNF90 colocalized with endogenous MAVS in mitochondria in HEK293T cells with or without poly (I:C) stimulation (**Figure 8C**). We further performed endogenous co-immunoprecipitation experiments, which indicated that RNF90 interacted with MAVS in untreated PMA-THP1 cells, and VSV infection enhanced the interaction at 4 h after infection (**Figure 8D**). Similar results were observed in VSV-infected HaCaT cells (**Figure 8E**). Next, we tried to figure out the region of MAVS responsible for its interaction with RNF90. As shown in **Figures 8F, G**, the residues aa180-360 of MAVS have the strongest association with RNF90, whereas aa 360-540 of MAVS lost the ability to interact with RNF90, suggesting the N-terminal of MAVS is responsible for interaction with RNF90. Finally, we mapped the binding regions on RNF90 for MAVS association. As shown in **Figures 8H, I**, the aa 1-166 and aa 324-511 of RNF90 exhibited no association with MAVS, whereas aa 167-511, aa 1-323, and aa 85-511 of RNF90 maintained the association with MAVS, suggesting aa 167-323 containing the coiled-coil structure might contribute to its interaction with MAVS. Altogether, our findings suggested RNF90 interacted with MAVS.

### RNF90 Promotes the Degradation of MAVS

**Figure 9:**
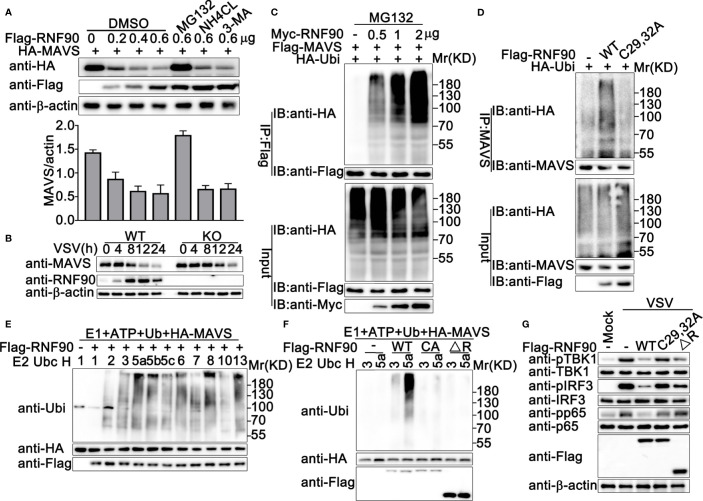
RNF90 enhances the degradation of MAVS **(A)** HEK293T cells were transfected with HA-MAVS and the increasing amounts of RNF90 plasmids as indicated. 24 h later, cells were treated with DMSO as control, MG132 (20 mM), NH_4_CL (5 mM), or 3-MA (2 mM) separately for 6 h and lysed for immunoblot assays (Top). The quantity of MAVS expression was normalized by β-actin (Bottom). **(B)** WT and RNF90-deficient (KO) HaCaT cells were infected with VSV (MOI = 1) for indicated time points, and the cell lysates were then subjected to immunoblot analysis. β-actin served as a loading control in all the immunoblot assays. **(C)** HEK293T cells were transfected with the indicated plasmids and increasing amounts of Myc-RNF90 (0, 0.5, 1, and 2 μg). 24 h later, cells were treated with MG132 (20 mM) for 6 h. Then, the cells were lysed and subjected to immunoprecipitation (IP) and immunoblot (IB) analysis. **(D)** HEK293T cells were transfected with HA-Ubiquitin (Ubi) plasmid, with empty vector (-), WT Flag-RNF90, or C29, 32A mutant plasmid. At 24 h after transfection, the cells were lysed and subjected to immunoprecipitation (IP) and immunoblot (IB) analysis. **(E, F)** Immunoblot analysis of MAVS ubiquitination *in vitro*. MAVS and WT RNF90 **(E)** or its mutants **(F)** were quickly translated *in vitro*, and the biotin-ubiquitin E1 and indicated E2s were added for the *in vitro* ubiquitination assays. Ubiquitination of MAVS was detected by anti-Ubi. **(G)** RNF90-deficient MEFs were transfected with indicated plasmids. At 24 h after transfection, the cells were infected with VSV (MOI = 1) for 4 h, and then the cell lysates were subjected to immunoblot analysis. The data are representative of three independent experiments.

Given that RNF90 interacted with MAVS, we next explored how RNF90 regulated the MAVS-mediated signaling pathway. The stability of MAVS was examined and the immunoblot analysis indicated that RNF90 overexpression inhibited MAVS expression in protein levels in cycloheximide (CHX) chase assay (**Figure S3**). This inhibition could be reversed by the proteasome inhibitor MG132, but not by NH4Cl or 3-MA (**Figure 9A**), suggesting RNF90 promoted MAVS degradation in a proteasome-dependent mechanism. Consistently, compared to WT cells, RNF90-deficient HaCaT cells exhibited a higher expression of MAVS upon VSV infection (**Figure 9B**).

### RNF90 Promotes the K48-Linked Ubiquitination of MAVS

Next, we investigated the effect of RNF90 on the ubiquitination of MAVS. RNF90 was co-transfected with MAVS and ubiquitin, and the immunoprecipitation and immunoblot analysis indicated that RNF90 overexpression increased the ubiquitination of MAVS in a dose-dependent manner (**Figure 9C**). RING-domain containing conserved cysteine and histidine residues is essential for the activity of RING-type ubiquitin-protein ligases (22). The C29, 32A mutant of RNF90, in which the integrity of the RING domain was destroyed (30), lost the ability to promote the ubiquitination of MAVS, suggesting the important role of the RING domain in the function of RNF90 (**Figure 9D**). Additionally, *in vitro* ubiquitination assays indicated that RNF90 enhanced the ubiquitination of STING directly (**Figure 9E**). Consistently, C29, 32A mutant of RNF90, or the RNF90 mutant lacking RING domain (ΔR) could not increase the ubiquitination of STING directly (**Figure 9F**). In addition, compared to WT RNF90, C29, 32A, and ΔR mutants exhibited less inhibition on the activation of TBK1, IRF3, and p65 upon VSV infection (**Figure 9G**).

**Figure 10:**
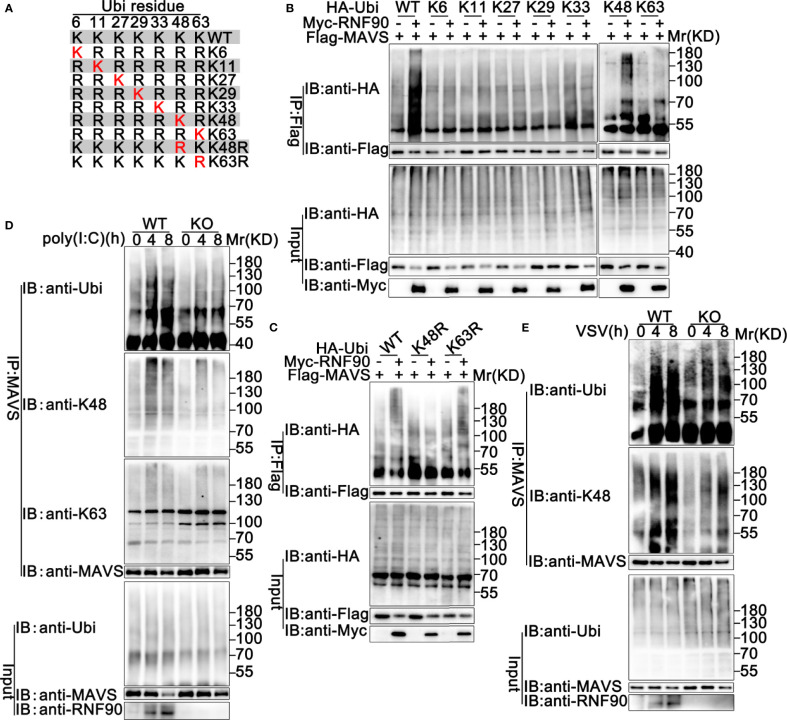
RNF90 promotes K48-linked ubiquitination of MAVS. **(A)** A schematic presentation of Ubi and its mutants. **(B, C)** HEK293T cells were transfected with the indicated plasmids. At 24 h after transfection, immunoprecipitation (IP) and immunoblot (IB) analysis were performed as indicated. **(D)** WT and RNF90-deficient (KO) HaCaT cells were stimulated with poly(I:C) (2.5 μg/ml) for the indicated time points. Then the cells were lysed and subjected to immunoprecipitation (IP) and immunoblot (IB) analysis. **(E)** WT and RNF90-deficient (KO) MEFs were infected with VSV (MOI = 1) for the indicated time points. Then the cells were lysed and subjected to immunoprecipitation (IP) and immunoblot (IB) analysis. The data are representative of three independent experiments.

Ubiquitin contains seven Lys residues (K6, K11, K27, K29, K33, K48, and K63) and seven different polyubiquitin chains can be generated (19). Ubiquitin mutants retaining only a single lysine residue were used to determine the type of linkage enhanced by RNF90 in the ubiquitination of MAVS (**Figure 10A**). As shown in **Figure 10B**, RNF90 promoted K48 mutants (only the Lys residue 48 was retained) mediated ubiquitination of MAVS, whereas showed no detectable effect on the ubiquitination mediated by the other six types of mutants, suggesting RNF90 promoted the K48-linked ubiquitination of MAVS. To further confirm the phenomenon, two mutants of ubiquitin, K48R (only the Lys residue 48 was mutated to Arg) and K63R (only the Lys residue 63 was mutated to Arg), were transfected into HEK293T cells with MAVS and RNF90. Immunoprecipitation and immunoblot analysis indicated that RNF90 promoted K63R mediated ubiquitination of STING, but not K48R, indicating the Lys residue 48 was essential to the RNF90-triggered linkage of MAVS with ubiquitin (**Figure 10C**). Furthermore, RNF90-deficiency in HaCaT cells inhibited poly(I:C) transfection induced K48-linked ubiquitination of MAVS, but no significant effect of RNF90 was observed on K63-linked ubiquitination of MAVS (**Figure 10D**). Additionally, RNF90-deficient MEF cells exhibited weaker K48-linked ubiquitination of MAVS upon VSV infection (**Figure 10E**) than WT cells. Altogether, our data indicated that RNF90 promoted the K48-linked ubiquitination of MAVS and its subsequent proteasome-dependent degradation.

## Discussion

In recent years, accumulating evidence has demonstrated the critical role of ubiquitination in regulating antiviral innate immune responses (44). Our previous research work suggested RNF90 negatively regulated DNA virus-triggered innate immune responses targeting STING (34). Because of the important role of STING in the RNA virus-triggered signaling pathway (45), it is reasonable for us to propose the hypothesis that RNF90 could act as a negative regulator during RNA virus infection. To prove our hypothesis, first of all, we explored whether RNF90 was expressed during RNA virus infection and our findings indicated that RNF90 expression was induced upon poly (I:C) transfection or RNA virus infection. Next, we evaluated the effect of RNF90 on RNA virus-induced immune responses in cells with RNF90 overexpression, RNF90-silenced cells, RNF90-deficient cells, and RNF90-deficient mice. Firstly, RNF90 overexpression in HEK293T cells inhibited IFN-β, CXCL10, and RANTES production upon poly (I:C) transfection or VSV infection. Secondly, in RNF90-silenced PMA-THP1 cells, VSV triggered antiviral innate immune responses was promoted, compared to PMA-THP1 cells transfected with control siRNA. Thirdly, we generated RNF90-deficient HaCaT cell line by CRISPR/Cas9 strategy, in which higher expression of IFN-β and CXCL10, enhanced activation of TBK1, IRF3, and p65, decreased expression of virus protein VSVg was observed upon VSV infection. Fourthly, we isolated and cultured the primary MEFs and BMDMs from WT and RNF90-deficient mice. Consistently, RNF90-deficient primary MEFs and BMDMs exhibited potentiated activation of TBK1, IRF3, and p65 and enhanced production of IFN-β, CXCL10, ISG56, and TNF-α after the stimulation of VSV or poly (I:C). Finally, RNF90-deficient mice exhibited prolonged survival and increased production of type I IFN and proinflammatory cytokine in the lung, liver, and spleen during VSV infection. All these data strongly demonstrated that RNF90 negatively regulated RNA virus-induced innate immune responses.

**Figure 11:**
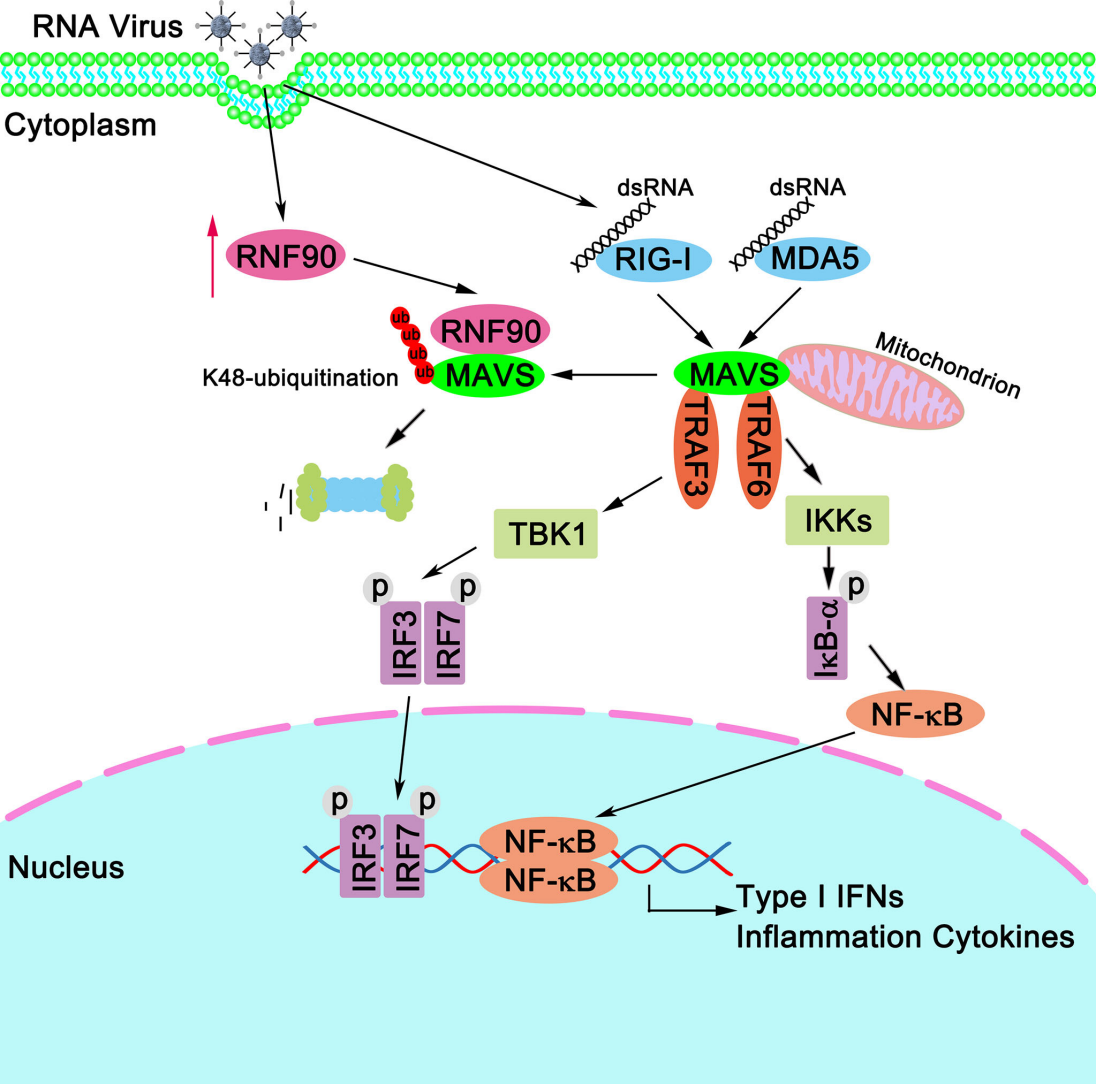
A schematic model illustrating functional involvement of RNF90 in MAVS-mediated antiviral signaling. Upon RNA virus infection, RIG-I binds to viral dsRNA and activates MAVS. MAVS recruits TRAF3 and TRAF6 to activate type I IFN and inflammation cytokines production. Host protein RNF90, as identified in this study, ubiquitinates MAVS for degradation and tones down the responses to RNA virus infection, thereby contributing to the maintenance of balanced antiviral innate immune responses.

Then, we tried to explore whether the effect of RNF90 on RNA virus-triggered innate immune responses was dependent on STING. Surprisingly, in STING-deficient THP1 cells, RNF90 knockdown still affected the production of IFN-β, CXCL10, and ISG56 upon poly (I:C) transfection or VSV infection, suggesting the effect of RNF90 on RNA virus-triggered antiviral immune response is independent of STING. Thus, during the negative regulation of RNA virus-triggered immune responses by RNF90, other target proteins regulated by RNF90 needed to be identified. Then, luciferase assays were performed to identify the downstream signaling and target protein of RNF90, revealing that MAVS might be the target protein. Next, both co-immunoprecipitation assays and confocal microscopy indicated RNF90 interacted with MAVS, and this interaction was enhanced by viral infection. Considering the essential role of MAVS in the RLR signaling pathway, it was reasonable that RNF90 might target MAVS for the negative regulation of innate immune responses against RNA viruses. Our previous data have demonstrated that RNF90 promotes the K48-linked ubiquitination on K150 and degradation of STING (34), so we first examined whether RNF90 had similar effects on MAVS. As expected, RNF90 increased the K48-linked ubiquitination and the subsequent degradation of MAVS in a proteasome-dependent mechanism, which relied on the RING domain of RNF90. However, we did not find the motif in the sequence of MAVS similar to that contained K150 in STING. Thus, as shown in **Figure 11**, our findings suggest a novel mechanism of negative regulation of antiviral innate immune response against RNA viruses.

Whereas the essential role of STING in DNA virus- or cytosolic DNA-triggered innate immune responses has been clarified (40–43), the function and mechanisms of STING in RNA virus-triggered antiviral immune responses remain less clear (46). It seems that STING regulates innate immune responses against RNA viruses in a virus- and cell-type-specific manner (45). For example, STING-deficient MEFs exhibited high susceptibility to VSV infection, but STING-deficient BMDCs or BMDMs did not (45). Furthermore, STING-deficient mice were defective in type I IFN production upon VSV infection but not encephalomyocarditis virus (EMCV), suggesting that STING may be only involved in RIG-I, but not MDA5-mediated signaling (45). Our research findings indicated RNF90 regulated RNA-triggered immune responses targeting MAVS, independent of STING, suggesting the negative regulatory role of RNF90 in the process of RNA virus infection may be universal. Thus RNF90 has the potential to manipulate the signaling pathways mediated by both RIG-I and MDA5.

Several molecules have been identified to target MAVS to regulate the antiviral signaling pathway (47). It is unclear why different molecules are needed to control MAVS degradation to turn off the antiviral innate immune responses. It will be very interesting to investigate these regulators’ expression patterns and functions during specific viral infections. Further investigations about the collaboration of these factors responsible for MAVS degradation in human diseases are also needed to address this issue.

In summary, our data suggest that RNF90, the expression of which is induced by the infection of RNA viruses, negatively regulates MAVS-mediated innate immune responses against RNA viruses. RNF90 interacts with MAVS, promotes its K48-linked ubiquitination and subsequent proteasome-dependent degradation. These results characterized a novel mechanism underlying the regulation and termination of MAVS-mediated innate immune responses against RNA viruses.

## Data Availability Statement

The raw data supporting the conclusions of this article will be made available by the authors, without undue reservation.

## Ethics Statement

The animal study was reviewed and approved by committee on animal care at Xinxiang Medical University (Approval Number: XXMUSPF2017-0045).

## Author Contributions

BY and JW designed the experiments, analyzed the data, and wrote the manuscript. BY, JW, YuL, YC, DS, GZ, SM, YaL, MC, and FC performed the experiments. HW helped with the revision of the manuscript. All authors contributed to the article and approved the submitted version.

## Funding

This work was supported by the National Natural Science Foundation of China Grants U1704183, 31970847, 32070949, and U2004103, Henan Medical Science and Technology Research Project LHGJ20200081, and by Henan Undergraduate Training Program for Innovation and Entrepreneurship S202010472024.

## Conflict of Interest

The authors declare that the research was conducted in the absence of any commercial or financial relationships that could be construed as a potential conflict of interest.

## Publisher’s Note

All claims expressed in this article are solely those of the authors and do not necessarily represent those of their affiliated organizations, or those of the publisher, the editors and the reviewers. Any product that may be evaluated in this article, or claim that may be made by its manufacturer, is not guaranteed or endorsed by the publisher.
