# Supplementary material for: Negative Regulation of RNF90 on RNA Virus-Triggered Antiviral Immune Responses Targeting MAVS
Source: Front Immunol. 2021 Aug 27;12:730483. doi: 10.3389/fimmu.2021.730483 (PMC8429505; doi:10.3389/fimmu.2021.730483)
Supplement: Supplementary file 1 [file DataSheet_1.docx]

Supplementary Material

## Supplementary Figures


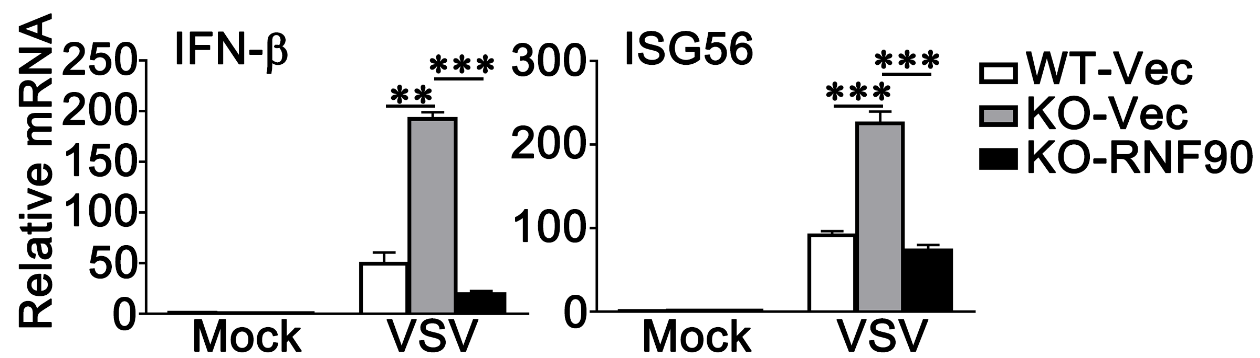


**Supplementary Figure 1** Wild-type (WT) and RNF90-deficient (KO) MEFs were transfected with indicated plasmids for 24 h, and then treated with VSV (MOI=1) for another 8 h. Then the cells were lysed for real-time PCR analysis. The data are representative of three independent experiments and are presented as mean ± SEM. ***p* < 0.01, ****p* < 0.001.


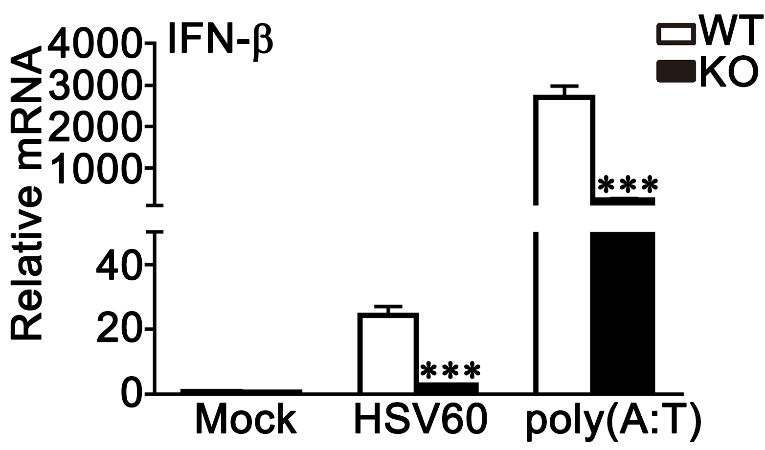


**Supplementary Figure 2** Wild-type (WT) and MITA-deficient (KO) PMA-THP1 cells were treated with HSV60 (1 μg/ml) or poly(dA:dT) (1 μg/ml) for 8 h. Then the cells were lysed for real-time PCR analysis. The data are representative of three independent experiments and are presented as mean ± SEM. ****p* < 0.001.


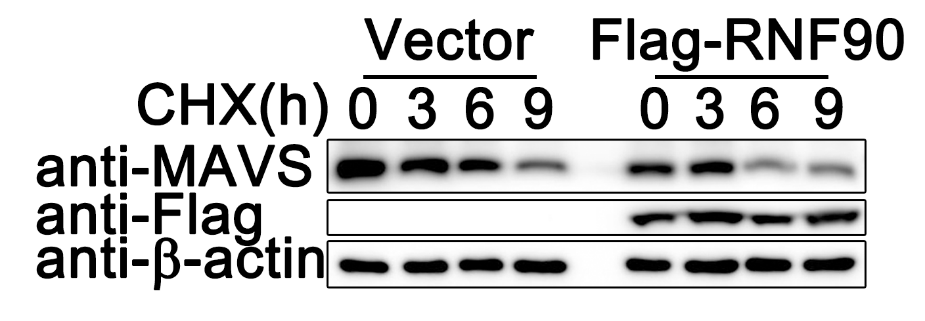


**Supplementary Figure 3** HEK293 T cells were transfected with indicated plasmid for 24 h and then treated with cycloheximide (CHX) (100 μg/mL) for indicated time points. Then the cells were lysed and subjected to immunoblot analysis. β-actin served as a loading control. The data are representative of three independent experiments.
